# A Systematic Review of Treatments of Post-Concussion Symptoms

**DOI:** 10.3390/jcm11206224

**Published:** 2022-10-21

**Authors:** Camille Heslot, Philippe Azouvi, Valérie Perdrieau, Aurélie Granger, Clémence Lefèvre-Dognin, Mélanie Cogné

**Affiliations:** 1 AP-HP, GH Paris Saclay, Hôpital Raymond Poincaré, Service de Médecine Physique et de Réadaptation 104, Boulevard Raymond Poincaré, 92380 Garches, France; 2 CESP, Inserm, Paris-Saclay University, UVSQ, 94807 Villejuif, France; 3 Rehabilitation Unit, Rennes University Hospital, 2 Rue Henri Le Guilloux, 35000 Rennes, France

**Keywords:** mTBI, post-concussion, rehabilitation, cognition, psychological care, physical

## Abstract

Approximately 10–20% of patients who have sustained a mild Traumatic Brain Injury (mTBI) show persistent post-concussion symptoms (PCS). This review aims to summarize the level of evidence concerning interventions for PCS. Following the PRISMA guidelines, we conducted a systematic review regarding interventions for PCS post-mTBI until August 2021 using the Medline, Cochrane, and Embase databases. Inclusion criteria were the following: (1) intervention focusing on PCS after mTBI, (2) presence of a control group, and (3) adult patients (≥18 y.o). Quality assessment was determined using the Incog recommendation level, and the risk of bias was assessed using the revised Cochrane risk-of-bias tool. We first selected 104 full-text articles. Finally, 55 studies were retained, including 35 that obtained the highest level of evidence. The risk of bias was high in 22 out of 55 studies. Cognitive training, psycho-education, cognitive behavioral therapy, and graded return to physical activity demonstrated some effectiveness on persistent PCS. However, there is limited evidence of the beneficial effect of Methylphenidate. Oculomotor rehabilitation, light therapy, and headache management using repetitive transcranial magnetic stimulation seem effective regarding somatic complaints and sleep disorders. The preventive effect of early (<3 months) interventions remains up for debate. Despite its limitations, the results of the present review should encourage clinicians to propose a tailored treatment to patients according to the type and severity of PCS and could encourage further research with larger groups.

## 1. Introduction

Approximately 42 million people worldwide and 150,000 in France are affected by an mTBI yearly [1,2,3]. According to the WHO Collaborating Task Force [4], the three main current criteria used to define mTBI are a Glasgow Coma Scale (GCS) score between 13 and 15 (30 min after the injury), a Post-Traumatic Amnesia (PTA) duration of less than 24 h, and/or a loss of consciousness lasting less than 30 min. After mTBI, 10–20% of the population will retain aftereffects called post-concussion Symptoms (PCS). According to the International Statistical Classification of Diseases and Related Health Problems (ICD-10), PCS is defined as a “Head injury usually sufficiently severe to result in loss of consciousness and then development within four weeks of at least three of the eight following symptoms: headache, dizziness, fatigue, irritability, sleep problems, concentration problems, memory disorders and emotion perturbations” [5]. Individuals who have sustained an mTBI have significantly more self-reported cognitive symptoms than controls up to 4 years after injury [6,7,8,9,10]. PCS is associated with anxiety and post-traumatic stress disorder (PTSD) in 16 to 26.8% of cases 6 months after mTBI [11,12,13] but other factors, such as vestibular/cervical dysfunction, may also contribute to outcomes [14]. Poor outcomes are difficult to predict at an individual level [10] but have been found to be statistically predicted by a combination of variables, including pre-injury factors (older age, lower education, pre-existing mental health problems), injury-related factors (assault-related injury, lower GCS score, presence of intracranial lesion), and early post-traumatic psychological factors (emotional distress, maladaptive coping) [6,15].

Several guidelines on the management of concussion/mTBI and PCS are available, created by expert groups, such as the Ontario Neurotrauma Foundation (https://braininjuryguidelines.org/concussion/ accessed on 15 October 2022) or the Centers for Diseases Control and Prevention (CDC; https://www.cdc.gov/traumaticbraininjury/mtbi_guideline.html accessed on 15 October 2022). Expert recommendations include early psycho-education, telephone counseling, graded return to physical activity, psychological treatment, cognitive rehabilitation, and vestibular or oculomotor rehabilitation [16]. However, despite the large number of publications in the field, the efficacy of interventions for PCS is still uncertain and remains a matter of debate (for a recent review, see ref. [16]). Moreover, PCS include a wide constellation of cognitive, psychological and somatic complaints, and it is difficult, based on the work published to date, to state which type of intervention is effective for which type of symptoms. A systematic review and meta-analysis on non-pharmacological interventions for persistent PCS was published recently and concluded that “based on very low to low certainty (…) the guideline panel found weak scientific support for commonly applied non-pharmacological interventions to treat persistent PCS” [16]. However, this latter review selected only 19 randomized controlled trials and did not include pharmacological interventions nor interventions related to specific symptoms associated with PCS, such as headache, fatigue, or associated mood disturbances.

The present review aims at summarizing the available evidence on the treatment of PCS after mTBI, including pharmacological interventions, and according to the type of symptom targeted (cognitive, physical, psychological). To this end, the proposed systematic review aimed at answering the following questions: Are pharmacological and non-pharmacological interventions, including early interventions, efficient in reducing PCS after mTBI? What are the risks of bias in the existing studies? Additionally, what is the level of quality of the evidence?

The review questions were defined using the PICO methodology: Population: adult participants with PCS after mTBI/concussion; Intervention: early intervention and all pharmacological and non-pharmacological interventions; Comparison: usual care, wait-list group, or any other intervention; Outcome: cognitive, physical, and psychological measures.

## 2. Materials and Methods

Study eligibility criteria. A systematic literature review was conducted in accordance with the PRISMA guidelines (www.prisma-statement.org accessed on 15 October 2022). We selected articles published in English until August 2021 and including treatment of adult individuals with PCS after mTBI. Previous literature reviews were not included, but studies quoted within these reviews were also included if relevant. Criteria for inclusion were the following: (1) intervention studies focusing on PCS after concussion/mTBI, (2) studies including a control group, and (3) studies focusing on adult patients (18 years or more). Studies including both mTBI and moderate to severe TBI were excluded (except if data regarding mTBI could be analysed separately). Studies including adolescents were included only if adolescent participants represented a minority of the study sample. For this review, we included only studies including a control group, but we chose to present studies matching our other inclusion criteria except for the control group in the Appendix A in order to contribute to our discussion. Both pharmacological and non-pharmacological interventions were included, and there was no specific time frame for intervention.

**Figure 1:**
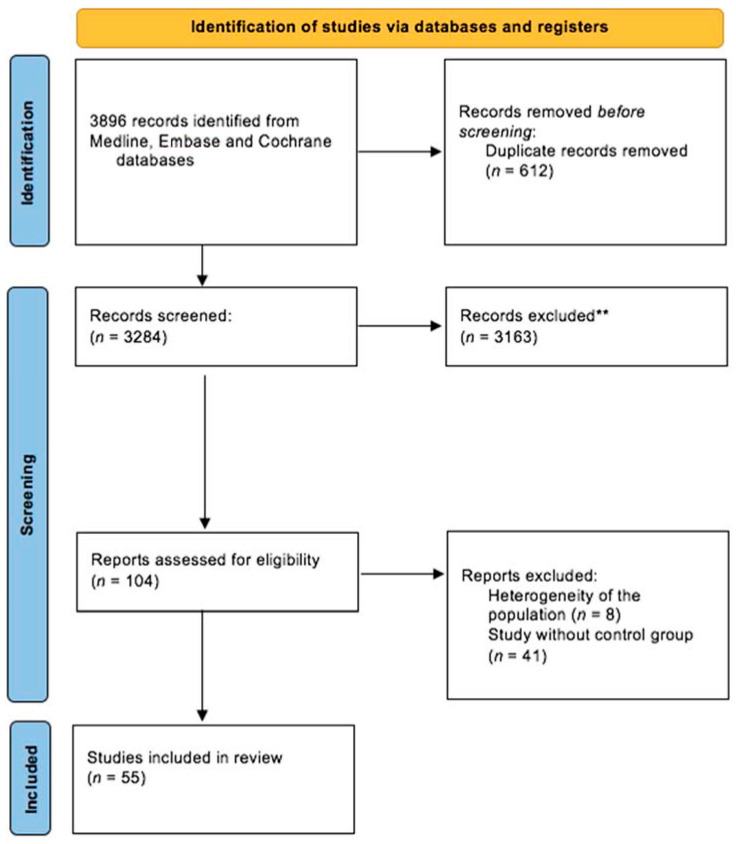
PRISMA Flow chart. ** main reasons for exclusion: studies conducted with children or adolescents, studies including a majority of participants with moderate to severe mTBI, and systematic reviews (screened for research of original articles).

Sources. The Medline, Cochrane, and Embase databases were searched. The keywords used were: «mild traumatic brain injury» AND «rehabilitation», «treatment», «post-concussion», «post commotional», OR «post-concussive». Then, we added the following keywords one by one: «attention», «memory», «dysexecutive», «executive», «neglect», «social cognition», «anxiety», «mood», «phobia», «post-traumatic stress disorder», «headache», «migraine», «irritability», «concentration», «insomnia», «sleep», «fatigue», «dizziness», «balance», OR «vestibular syndrome». Articles were then independently selected on the basis of title and abstract screening by two authors (CH and MC). If there was a disagreement between these two authors, a third author (PA) intervened to reach a consensus. The following flow chart (Figure 1) illustrates the article selection procedure.

Data extraction and quality assessment. Studies were divided into four groups according to the main target or timing of intervention: cognitive symptoms and PCS in general, mood and sleep disorders, somatic complaints and fatigue, and early interventions (within three months since injury). Information extracted (Table A1, Table A2, Table A3 and Table A4) included: experimental design, age and gender of the participants, etiology of mTBI, time since injury, the main objective of the study (including nature of the intervention and of control treatment when applicable), the number of participants, outcomes and tools, and the main results. Quality assessment was based on the Incog grading system: [17] A = Recommendation supported by at least one meta-analysis, systematic review, or randomized controlled trial of appropriate size with a relevant control group; B = Recommendation supported by cohort studies that at minimum have a comparison group, well-designed single-subject experimental designs, or small sample size randomized controlled trials; C = Recommendation supported primarily by expert opinion based on their experience, or uncontrolled case series without comparison groups. The risk of bias in the included studies was assessed by two authors (MC and PA) using the Cochrane Rob2 tool revised version [18]. This tool considers five domains that can be a source of bias: randomization process, deviations from intended interventions, outcome data, measurement of the outcome, and selection of the reported results. Each domain is assessed as having a low, high, or unclear risk of bias. Upon judgment of the risk of bias for each domain, the authors judged the overall risk of bias for each publication included.

Studies selected (Figure 1). First, 3896 abstracts were identified using the keywords «mild traumatic brain injury» (in the title or abstract) AND «rehabilitation», «treatment», «post-concussion», «post commotional», OR «post-concussive» (in all fields). Regarding cognitive symptoms after mTBI, 1399 records were identified by adding the following keywords one by one: “attention”, “memory”, “dysexecutive”, “executive”, “neglect”, “concentration”, OR “social cognition”. Lastly, 18 studies were included. Regarding psychological symptoms, 1626 records were identified by adding the following keywords one by one: “anxiety”, “mood”, “affect”, “phobia”, “phobic”, “insomnia”, “sleep”, “irritability”, OR “post-traumatic stress disorder”. In the end, four studies were included and all of them concerned sleep disorders. Regarding physical symptoms after mTBI, 871 records were identified by adding the following keywords one by one: “fatigue”, “dizziness”, “balance”, “vertigo”, “headache”, «migraine», OR “vestibular syndrome”. Finally, 15 studies were included. Regarding early interventions after mTBI, we selected from 18 articles from previous research related to intervention in a delay of 3 months or less after mTBI.

Overall, 104 articles matched our inclusion criteria and were selected. Eight of these papers were excluded as participants were not restricted to patients with mTBI (some of them included moderate or severe TBI or patients with PTSD without TBI). The main manuscript will only focus on papers rated as A or B level of evidence (55 articles). The remaining 41 articles (uncontrolled group studies or isolated case reports) are presented as Appendix A. The main characteristics of the 55 selected studies are shown in Table A1, Table A2, Table A3 and Table A4, with their corresponding level of evidence.

## 3. Results

### 3.1. Treatments Focusing on Cognitive Symptoms and the Reduction of PCS in General

Overall, we selected 18 Grade A or B studies that focused on cognitive complaints after mTBI and/or reduction of PCS in general, based on different methods. Different methods such as cognitive training, psycho-education, pharmaceutical treatments, non-invasive brain stimulation, hyperbaric oxygen, technology-assisted rehabilitation, and others, were proposed.

#### 3.1.1. Cognitive Training Programs and Psycho-Education

Five Grade A or B randomized controlled trials (RCT) based on cognitive rehabilitation and/or psycho-education were selected. All of these trials found positive results. One of them, found in ref. [19], included 89 participants in an interdisciplinary 22-week program (S-REHAB) with exercise therapy and physiotherapeutic coaching, showing a reduction of PCS measured with the Rivermead Post-Concussion Symptoms Questionnaire (RPQ) immediately post-treatment and 6 months later. A 10-week program utilizing group-based compensatory cognitive training in 119 veterans also found a significant improvement in attention, learning abilities, and executive functioning [20]. Tiersky et al. [21] combined cognitive remediation and psychotherapy for 20 participants with mTBI and PCS lasting more than one year (including two patients with moderate TBI) and found a significant reduction of anxiety and depression and an improvement in divided attention at 1 and 3 months follow-up. However, there was no significant improvement in community integration scores. Another study [22], including 112 participants, showed that an 8-week interdisciplinary, individually tailored intervention based on a gradual return to activities and principles from cognitive behavior significantly reduced PCS at 3 months. Novakovic-Agopian et al. [23] found some efficacy in a goal-oriented attentional self-regulation training program performed during 5 weeks by 40 veterans with comorbid PTSD and mTBI on cognitive functions, emotional regulation, and functional performance. Nevertheless, another study [24] compared different cognitive rehabilitation interventions (psycho-education, computer-based training, therapist-directed manualized training, and integrated therapist-directed training combined with cognitive-behavioral psychotherapy) but did not find any difference between techniques concerning the primary outcome criterion, the Paced Auditory Serial Addition Test. In conclusion, most of these five RCTs reported significant positive effects on cognitive functions, although there is no evidence that one single program is more effective than the other. In addition, three uncontrolled studies reported positive effects of various cognitive interventions, including Attention Process Training-II or Goal Management Training (see Appendix A).

#### 3.1.2. Pharmacological Treatments

Five Grade A or B publications reported the positive effects of three drug treatments (Guanfacine, Methylphenidate, and Enzogenol) on cognitive symptoms after mTBI, but three of these articles were based on the same trial at different time-points [25,26,27]. 

One of them [28] found an improvement in working memory (evaluated by an “N-back” task) and increased activation of the frontal lobe in functional MRI 2.5 h after the administration of alpha2-antagonists (Guanfacine) in 13 patients. Another [25] found a positive effect of Methylphenidate on fatigue and processing speed (but not on pain) with a dose-dependent effect in 51 patients (including 4 with moderate TBI). They followed 30 participants who had reported positive effects during the initial phase of this latter study and were treated with methylphenidate for a further 6 months and still showed significant improvement, as compared to baseline, on mental fatigue, depression, anxiety, processing speed, attention, and working memory [26]. A follow-up study was then conducted by the same group over a period of approximately 5.5 years in 17 patients [27]. A comparison was made between those who had continued or discontinued Methylphenidate. Treatment was associated with improvement in mental fatigue, depression, anxiety, and processing speed, suggesting that the Methylphenidate effect is maintained over time but reversible if discontinued [27]. Lastly, administration of Enzogenol (pine extract) during 6 weeks significantly reduced self-perceived cognitive impairments (evaluated with the Cognitive Failures Questionnaire) in 62 participants [29].

To summarize, five studies using pharmaceutical treatments reported positive results on the outcome (mainly with Methylphenidate). In addition, one uncontrolled study found a beneficial effect of antidepressant treatment (see Appendix A).

#### 3.1.3. Non-Invasive Brain Stimulation

Only one randomized controlled trial was selected in this category, but five additional uncontrolled studies were reported (see Appendix A). Moussavi et al. [30] found that participants who had an injury < 12 months who received active rTMS (repetitive Transcranial Magnetic Stimulation) showed significant improvements in the RPQ compared to those in the same subgroup who received sham stimulation and to those with a longer duration of injury (>14 months) who received active rTMS.

#### 3.1.4. Hyperbaric Oxygen Therapy

Five Grade A randomized controlled trials evaluating the effect of hyperbaric oxygen (HBO2) treatment on cognitive functions in patients with mTBI found contradictory results (two positive, three negative). Harch et al. [31] found that 63 participants with 150 Kpa HBO2 significantly improved on a wide range of measures, including cognition, mood, PCS, sleep, and quality of life. In another study including 56 patients [32], HBO2 improved cognitive functions and quality of life. However, three other randomized sham-controlled trials [33,34,35] did not find any cognitive improvement after HBO2 in a large sample of participants suffering from PCS after mTBI. 

In conclusion, overall, these studies do not support the use of HBO2 at this time for persistent PCS after mTBI. 

#### 3.1.5. Technology-Assisted Cognitive Rehabilitation

Two studies were selected, one positive and one negative. Belanger et al. [36] did not find any significant effect of a web-based educational intervention regarding TBI (severity, symptoms and their management, expectation of recovery) on symptom severity or occurrence in 158 participants. However, it should be noted that subgroup analyses suggested some benefit in the group of patients receiving concurrent mental health treatment at baseline who reported significantly less PCS than controls at 6 months. Cooper et al. [24] found an improvement in auditory attention assessed using the Paced Auditory Serial Addition Test after several interventions (psycho-education, computer-based Cognitive Réhabilitation (CR), therapist-directed manualized CR, and integrated therapist-directed CR combined with cognitive-behavioral psychotherapy) in 126 participants. 

In addition, four uncontrolled studies were found, assessing the effect of computerized attentional training, a self-run computer-based psycho-education program, and a videophone-based therapy combined with psycho-education or assistive technology aids (such as IPads with scheduling application, iPhone with a sample voice memo, or electronic list), with mixed results (see Appendix A).

#### 3.1.6. Other Rehabilitation Techniques

Five uncontrolled studies were included in this category, including diverse forms of intervention, such as mindfulness-based stress reduction, a targeted treatment on specific symptoms/impairments (such as psychological, sleep, ocular, vestibular symptoms), head-eye vestibular motion therapy, musical training, or Qigong practice (see Appendix A). Although positive findings were reported, the level of evidence within the studies found was very low.

### 3.2. Treatments Focusing on PTSD, Mood, and Sleep Disorders

We selected four studies (three Grade A) that specifically focus on sleep disorders, with mixed results.

In addition, we found six studies specifically addressing PTSD after mTBI with encouraging results, but they were all uncontrolled studies (see Appendix A).

A large (*n* = 356) study using telephone-based problem-solving treatment [37] found a significant improvement in the sleep quality of participants with mTBI at 6 months, but the effect was not maintained 12 months post-injury. Another study [38] showed that cognitive behavioral therapy significantly reduced sleep disturbance with a moderate effect in 24 participants after a mild to moderate TBI. However, there were no significant group differences in objective sleep quality, cognitive functioning, post-concussion symptoms, or quality of life, possibly due to a lack of statistical power. Furthermore, 6-week morning blue-light therapy compared to placebo light in 35 adults with an mTBI under 18 months showed a significant improvement in sleep, daytime sleepiness, depression, PCS, and executive functions [39,40].

### 3.3. Treatment Focusing on Somatic Complaints and Fatigue

We selected 14 studies in this category (six Grade A). 

#### 3.3.1. Balance Disorders

The treatment of balance disorders and vertigo was evaluated in four controlled studies and in nine uncontrolled or case-series studies (see Appendix A). Kleffelgaard et al. [41] reported on the efficacy of 8-week group-based vestibular rehabilitation intervention in 65 participants, which appeared to speed up recovery for patients with dizziness and balance problems. However, the benefits were not maintained 2 months after the end of the intervention. A randomized study of 21 patients suffering from balance deficit after mTBI lasting more than 12 months evaluated the effect of video game therapy on the X-Box 360 console compared to balance platform therapy [42]. It showed that both groups improved in Community Balance and Mobility Scale scores, but only the video game therapy group improved on the Unified Balance Scale and Timed Up and Go test. Schneider et al. [43] found some efficacy in a combination of 8-week vestibular rehabilitation and cervical spine physiotherapy with a decrease in the time until medical clearance to return to sport in individuals with prolonged PCS at the subacute stage. Lastly, a study of 71 patients found some efficacy of 40 sessions over 12 weeks of hyperbaric oxygen therapy on balance impairments [44].

In summary, there is some, although limited, evidence to suggest that specific training may improve dizziness and balance disorders after mTBI.

#### 3.3.2. Headache and Migraine

The management of post-traumatic headaches or migraines after mTBI was evaluated in seven controlled studies and five uncontrolled studies (see Appendix A).

Four controlled studies focused on the effect of rTMS on headaches with mostly positive results. Leung et al. reported two positive studies of rTMS applied on the left motor cortex [45] or on the left dorsolateral prefrontal cortex [46] to alleviate post-traumatic headache (prevalence and pain intensity) 1 and 4 weeks post-treatment. Another study, including 20 participants, used 10 sessions of rTMS therapy applied to the left dorsolateral prefrontal cortex with mixed results, with effects which fell below clinical significance thresholds [47]. Choi et al. [48] found that 10 sessions of rTMS applied to the primary motor cortex of the affected hemisphere significantly reduced pain intensity after treatment and after 1, 2, and 4 weeks. Even if large randomized controlled trials seem necessary to confirm these preliminary results, rTMS seems promising for reducing headaches in mTBI with PCS.

Kjeldgaard et al. [49] found no significant effect of 9 weeks of cognitive behavioral therapy on 90 participants for headache and pain perception in comparison to a waiting list group. Two sessions of manual therapy on the neck were tested in a small RCT (*n* = 23) in comparison to cold packs on the neck. Treatment was associated with a significant reduction of pain index 2 weeks after the end of treatment, but this effect was no more statistically significant 5 weeks later [50]. Esterov et al. (2021) [51] showed a reduction in pain in 26 participants assessed using a visual analogic scale after an osteopathic manipulative treatment, but no significant difference regarding pain was found within the questionnaire results. 

In addition, five uncontrolled studies evaluated the drug treatment of post-concussive headaches with drugs such as botulinum toxin, gabapentin, tricyclics, epidural injection of saline, and oxygen or monoclonal antibodies targeting Calcitonin gene-related peptide, but the level of evidence of these studies remains very low (see Appendix A).

#### 3.3.3. Oculomotor Disorders

The treatment of oculomotor and vision disorders after mTBI was addressed in three controlled studies with mixed results (and in several additional uncontrolled studies, as can be seen in Appendix A). Thiagarajan et al. reported in two separate papers the results of a 6-week cross-over trial comparing oculomotor rehabilitation with placebo training in 12 patients with near-vision symptoms [52,53]. During each session, all three oculomotor subsystems (vergence/accommodation/version) were trained. They found an improvement in vergence and of near work-related symptoms and visual attention [52], and of oculomotor control, reading rate, and overall reading ability [53]. Hyperbaric oxygen therapy did not show any effect on eye-tracking abnormalities in 60 patients with mTBI compared to a sham-control treatment [54]. 

In summary, although some preliminary positive results have been reported, the level of evidence of oculomotor rehabilitation remains low.

#### 3.3.4. Post-Injury Fatigue

We found only one Grade A study. Kolakowsky-Hayner et al. [55] included 123 patients to evaluate the effect of a graduated physical activity program (home-based walking program using a pedometer to track a daily number of steps accompanied by tapered coaching calls over a 12-week period) compared with a control condition (nutritional counseling with the same schedule of coaching calls). The results showed less reported fatigue at the end of the active part of the intervention (24 weeks) and after a wash-out period (36 weeks).

### 3.4. Early Interventions

Eighteen controlled studies assessed the effect of an early intervention (<3 months post-injury) designed to prevent the occurrence and chronicity of PCS. However, there were mixed findings.

Seven publications reported positive results. Mittenberg et al. [56] showed that based on a printed manual and a consultation with a therapist (providing psycho-education, information, techniques for reducing symptoms, and instructions for a gradual return to premorbid activities), significantly shorter symptom duration, fewer symptoms, fewer symptomatic days, and lower severity levels were found in 58 participants after very early intervention (prior to hospital discharge). Ponsford et al. [57] found that in 202 participants, there were positive effects of providing an information booklet outlining the symptoms associated with mTBI and suggestions of coping strategies one week after the injury. Three months post-injury, patients in the intervention group reported significantly fewer symptoms and were less stressed than those in the control group. Bell et al. [58] showed the positive impact of focused, scheduled telephone counseling (five phone calls) on the mean symptom score of 366 participants, reducing the proportion of patients reporting each individual symptom (except anxiety) and issues with daily functioning. Caplain et al. [59] proposed that for 80 participants in the early stage post-injury (<1 month) presenting high risk factors for persistent PCS, multidimensional intervention with 14 sessions combining psycho-education and cognitive rehabilitation was desirable. They found a significantly decreased risk of persistent PCS at the 6-month follow-up. The preventive effect of early CBT (<6 weeks) was found to be effective by Silverberg et al. [60] in 28 patients, decreasing the risk of persistent PCS at 3 months. The effect size on PCS reduction was moderate. The use of a text messaging-based education and behavioral support was associated with a non-significant trend for a decreased report of irritability, anxiety, headaches, and concentration difficulties within 14 days post-trauma (n = 43) [61]. One quasi-experimental non-randomized study found that social work intervention (providing reassurance and education regarding the recovery process and follow-up guidelines, including brief alcohol intervention) at the acute stage post-mTBI at the emergency department significantly reduced alcohol use 3 months post-injury [62].

However, contrasting results were also reported in nine publications. No significant difference was found in self-reported symptom severity between bed rest and no bed rest for 107 patients during the first 10 days after mTBI [63]. Ghaffar et al. [64] included 191 participants within one-week post-injury who were randomly assigned to multidisciplinary treatment or no treatment. They did not find any group difference at 6 months (although, in subjects with a psychiatric history, the provision of treatment was associated with significantly fewer depressive symptoms). Andersson et al. [65] offered an early individualized, tailored multidisciplinary outpatient rehabilitation program involving physiotherapists, occupational therapists, and social workers after mTBI. Patients had repeated outpatient appointments and thereafter, telephone contacts. There was no significant difference between the control group one year post-injury. Heskestad et al. [66] assessed the effect of very early (2-week post-injury) educational intervention based on one single consultation focusing on cognitive counseling, advice, information, and reassuring. They failed to find any significant difference compared to the control group regarding symptoms, depression, sleep, and fatigue at 3- and 6-month follow-ups, but there was a very high dropout rate (85%). An early intervention visit (14 to 21 days after the trauma) in addition to written information and treatment did not show more efficacy than treatment alone on symptom level at 3 months in a study including 97 patients [67]. Vikane et al. [68] did not find any significant between-group differences regarding the 12-month return to work following a multidisciplinary outpatient follow-up program for patients being at-risk or sick-listed with persistent PCS. However, there were fewer post-concussion symptoms in the intervention compared to the control group at 12 months. Varner et al. [69] found no beneficial effect of an intervention within 24 h post-injury based on cognitive rest and graduated return to usual activity discharge instructions (n = 118). Post-commotional symptoms were not significantly different in the intervention group compared to controls 2 and 4 weeks post-injury. Early intervention (cognitive behavioral intervention with psycho-education on mTBI and enhancement of the sense of self-control) compared to telephone counseling in “at risk” patients failed to find any significant difference in return-to-work anxiety and depression [70]. Paradoxically, in this latter study, telephone counseling was associated with fewer complaints and more frequent full recovery at 12-month follow-up than cognitive behavioral intervention. Audrit et al. [71] did not find any significant time x group interaction of a psycho-educative and counseling intervention (SAAM) on PCS assessed using the RPQ.

In addition, two early drug trials may be mentioned here. Early Cerebrolysin (a nootropic drug which has been found useful to improve cognitive function in patients with Alzheimer’s disease) therapy (within 24 h) improved cognitive functions, especially long-term memory and visuo-constructive functions in 32 patients 3 months after mTBI [72]. However, Atorvastatin, which was assumed to improve cerebral plasticity, was administered for 7 days in 52 patients with mTBI at an early stage (within 1 week) but did not show any significant effect on PCS (evaluated by the RPQ) 3 months later [73].

In summary, it is difficult to conclude at this stage as results differ from one study to the other. Prolonged bed rest should not be recommended, but the beneficial effect of an early educational or multidisciplinary intervention remains to be debated.

### 3.5. Risk of Bias Assessment

**Figure A1:**
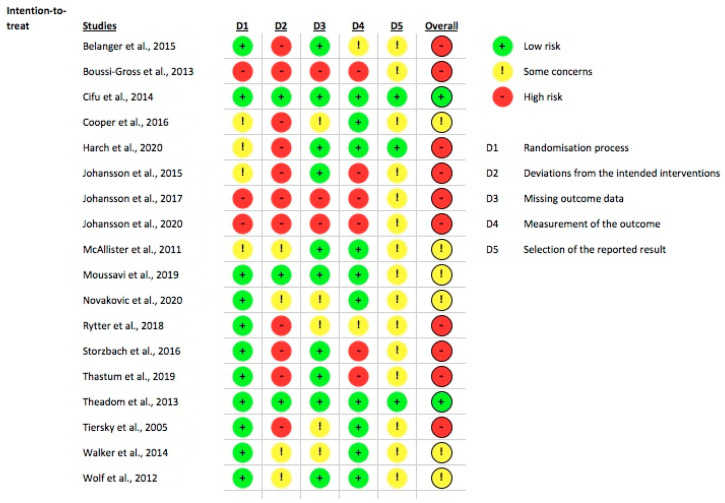
Risk of Bias for each study included in the Section 3.1, assessed using the Cochrane Rob2 tool revised version [19,20,21,22,23,24,25,26,27,28,29,30,31,32,33,34,36,80].

**Figure A2:**
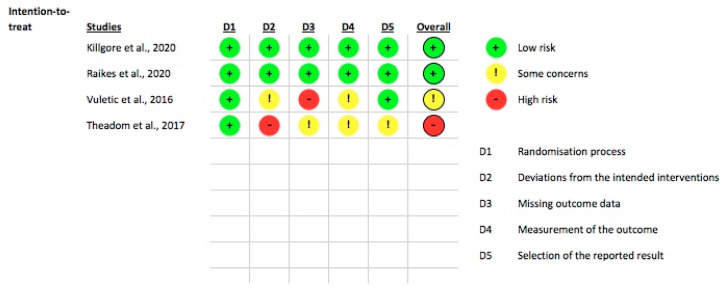
Risk of Bias for each study included in the Section 3.2, assessed using the Cochrane Rob2 tool revised version [37,38,40,44].

**Figure A3:**
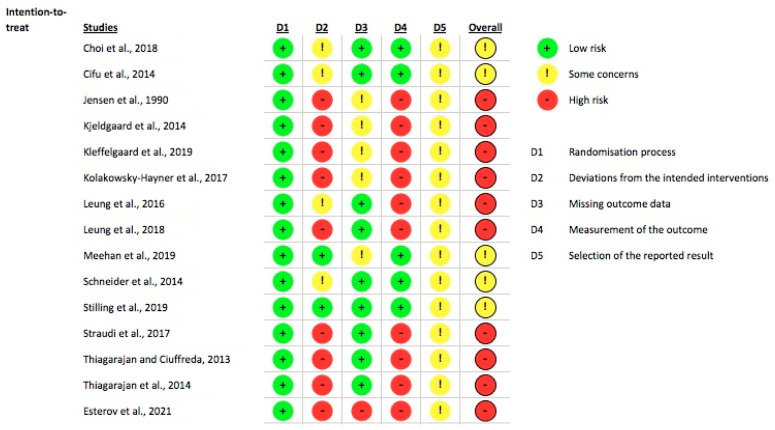
Risk of Bias for each study included in the Section 3.3, assessed using the Cochrane Rob2 tool revised version [35,41,42,43,44,45,46,47,48,49,50,51,52,53,55].

**Figure A4:**
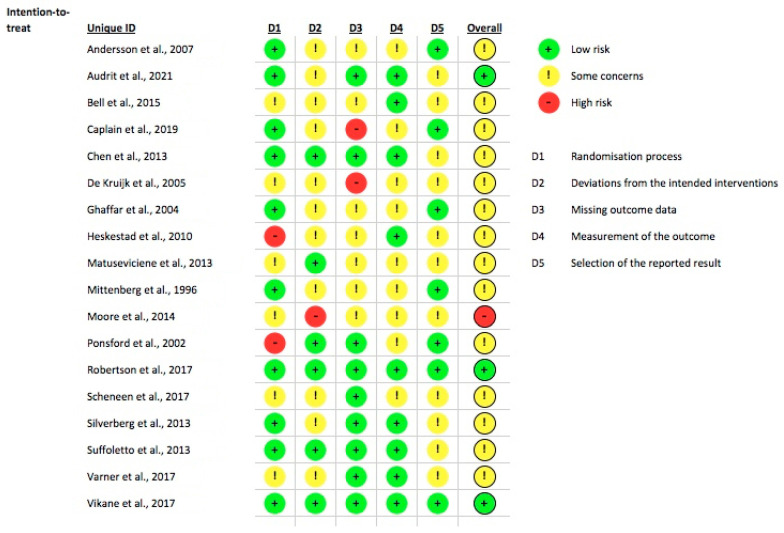
Risk of Bias for each study included in the Section 3.4, assessed using the Cochrane Rob2 tool revised version [56,57,58,59,60,61,62,63,64,65,66,67,68,69,70,71,72,73].

Among the 55 RCT selected, 22 studies were judged to have an overall high level of risk of bias, 26 studies raised some concerns regarding the risk of bias, and only 7 studies had a low risk of bias (please consider Figure A1, Figure A2, Figure A3 and Figure A4 in Appendix B).

## 4. Discussion

The objective of the present systematic review was to identify therapeutic approaches which may improve persistent PCS after mTBI.

### 4.1. Treatment of Cognitive Symptoms and Reduction of PCS in General

Among the five studies based on cognitive training and/or psycho-education, four reported positive results, which are quite encouraging and constitute a relatively good level of evidence to recommend such training in patients with persistent PCS, including those with associated PTSD. However, the level of evidence of alternative treatments, such as pharmaceutical drugs, non-invasive brain stimulations (in particular rTMS), hyperbaric oxygen, or technology-assisted rehabilitation, remains low or inconsistent, and none of these approaches can be recommended for clinical practice at this stage, even if some promising results have been found with rTMS.

### 4.2. Treatment of PTSD, Mood, and Sleep Disorders

Regarding mood disorders and PTSD, we could not find any controlled study specifically addressing these issues in participants with mTBI, but positive findings were reported in a few uncontrolled studies (see Appendix A) or in samples including, but not limited to, mTBI. For example, Ponsford et al. [74] found some efficacy in a 9-week CBT for reducing anxiety and depression in 75 participants after mild to severe TBI. Four controlled studies focusing on sleep disorders were selected, with encouraging but contrasting results, so the level of evidence remains modest. Cognitive-behavioral therapy and psychological support were found to be useful in improving sleep quality after mTBI in two studies, including one large randomized controlled trial [37,38]. Light therapy also seems promising for improving sleep quality after mTBI [39,40].

### 4.3. Treatment of Somatic Complaints, Headaches, and Fatigue

Vestibular rehabilitation, cervical spine therapy, or techniques based on video games and virtual reality were found to improve balance disorders in four controlled studies, including two Grade A RCTs [41,42,43]. However, the persistence of a beneficial effect after the end of treatment seems questionable [41]. rTMS was found to be efficient for post-traumatic headaches after mTBI in three randomized sham-controlled studies [45,46,48] but one of them found effects which fell below clinical significance thresholds [47]. Thus, the level of evidence is low. Other interventions targeting post-traumatic headaches after mTBI, such as cognitive behavioral therapy [49] or manual therapy of the neck [50], reported negative results. Among three controlled studies targeting oculomotor disorders, an improvement was found regarding oculomotor rehabilitation in two studies by the same group [52,53], but hyperbaric oxygen treatment had no significant effect on eye movement disorders [54]. Finally, one Grade A RCT found that fatigue can be significantly alleviated by a graduated physical activity program [55].

### 4.4. Early Interventions

Eighteen controlled studies assessing the effect of an intervention within the first 3 months after the injury to prevent the occurrence or the persistence of PCS were selected; however, these studies provided mixed findings. Eight studies reported positive results with early interventions based on various combinations of providing written and oral information, reassurance, psycho-education, counseling (in person or by telephone or texting), and/or CBT [22,56,57,58,59,60,61,62]. In contrast, nine other early intervention studies failed to determine a beneficial effect. Some of these studies used multidisciplinary rehabilitation [64,65,68], and others relied on only one single early consultation [66,67,69] or on bed rest [63]. However, two well-designed RCTs based on multidimensional psycho-education, counseling, or CBT reported negative results [70,71]. It is thus difficult to draw any firm conclusions from these findings, although quite encouraging results were reported to suggest some beneficial preventive effects of a combination of information, psycho-education, reassurance, counseling, and CBT at the very early stage after mTBI.

In summary, the results of this review suggest that different rehabilitation programs, particularly cognitive training, psycho-education, telephone counseling, but also graded physical activity, could be efficient in decreasing persistent PCS of adult patients after mTBI. These results, although heterogeneous, support the use of a range of treatments for persistent PCS after mTBI and thus could provide guidance for healthcare professionals in the management of these patients and steer future studies. The present findings should encourage the development of evidence-based guidelines and information for patients, caregivers, and health professionals to improve global outcomes.

More precisely, it appeared that specific treatments could be useful to target different specific symptoms. In patients with predominant cognitive and global complaints, with or without associated PTSD, a combination of cognitive training and psycho-education could be useful. Anxiety and depression post-mTBI may be reduced using CBT. Sleep disorders may be improved by CBT or blue light therapy. Balance disorders could be at least temporarily improved by vestibular rehabilitation. Post-traumatic headaches could be reduced with rTMS. Fatigue can be alleviated by a graduated physical activity program. Finally, at-risk patients seen at the early stage (<3 months) could benefit from a program including psycho-education, reassurance, counseling, and/or CBT. In opposition, hyperbaric oxygen therapy, pharmaceutical drugs, and rTMS (for symptoms other than headaches) should not be recommended, given the available evidence.

The main strength of this review, based on 55 controlled studies (including 35 rank A), is the fact that it is, to the best of our knowledge, the first systematic literature review conducted according to the PRISMA guidelines on a large spectrum of PCS after mTBI, performed on several databases (Medline, Embase, and Cochrane), including research published up until August 2021. Other reviews have been published previously on specific aspects of PCS. For example, recent reviews [75,76,77] focused on the effect of physical exercise in patients with persistent PCS and found that exercise significantly reduced the severity of PCS, the percentage of patients with PCS, and days off work, as compared to controls. Other recent reviews addressed issues such as interventions in sport-related concussion [17,78], reporting evidence in support of cervical rehabilitation, vestibulo-ocular rehabilitation, aerobic exercise, or rTMS [51,79], suggesting promising preliminary results for the treatment of post-concussive depression and headaches. As previously mentioned, a systematic review with meta-analysis provided only very low to low levels of evidence to support commonly applied non-pharmacological interventions for persistent PCS [16]. The objective of the present study, in comparison with previous reviews, was to present a broad overview of the different possible interventions on the different facets of PCS rather than focusing on one single symptom or patient population.

However, this study has some limitations. First, there is probably a publication bias, as studies with negative results are often unpublished. We tried to minimize this bias by extending our search to databases other than Medline. In addition, several selected studies had a lower level of evidence, mainly because of the low number of participants included. Only seven studies had a low risk of bias with contrasting results, thus limiting the overall level of evidence, with the exception of blue light therapy, for which two low-risk of bias studies reported positive results. Furthermore, the studies were extremely heterogeneous in terms of rehabilitation type, the primary endpoint, outcome measures, time after trauma, and the number of patients; hence, meta-analysis was impossible, and this heterogeneity limits the potential conclusions. We did not find enough studies evaluating the impact of rehabilitation at a very long term (several years) after the mTBi; thus, we were unable to determine the maximum period of time during which rehabilitation could have a positive impact on persistent PCS. A final limitation is related to the wide variation in population samples and/or injury mechanisms included in the different studies in the present review (such as civilians, veterans, athletes, etc.). It was unfortunately not possible to untangle the effects of intervention in these different subpopulations, but this could be an important issue to consider in future research.

## 5. Conclusions

Despite these limitations, the results of the present review should encourage clinicians to propose tailored treatment to patients with persistent PCS, according to the type and severity of symptoms. For example, cognitive training and psycho-education could be recommended for patients with cognitive complaints and persistent PCS, cognitive behavioral therapy and light therapy for sleep disorders, rTMS for post-traumatic headaches after mTBI, a graduated physical activity program for persisting fatigue, and early counseling, reassurance, psycho-education, and/or CBT could be recommended for at-risk patients at an early stage (<3 months). Further research should be encouraged to assess such programs in larger groups of patients using a randomized controlled design.

## Data Availability

Not applicable.

## Appendix A

Table A1, Table A2, Table A3 and Table A4 are the presentation of the 55 articles included in this systematic literature review.

**Table A1:** Cognition and PCS (18 articles).

First Author, Year of Publication	Study Design	Age (Mean) and Gender of the Participants	Etiology of mTBI	Delay between mTBI and Inclusion	Main Aim	Number of Participants	Outcomes and Tools	Results	+ If Main Result Is Significant, − If Not	Incog Recommendation Level
Belanger et al., 2015 [36]	RCT	18–25 y.o. = 14.6% 26–35 y.o. = 39.9% 36–45 y.o. = 36.7% 46–55 y.o. = 8.9% 75% male	Not described	<2 years (37% <1 month, 31.5% = 1 month–1 year, 31.5% > 1 year	To investigate the effectiveness of a web-based educational intervention for reducing post-concussion symptoms.	158	-Cognition: Neurobehavioral Symptom Inventory, Brief-Symptom Inventory-18, Self Efficacy for Symptom Management Scale -PTSD: PTSD Checklist Civilian Version -Other: Questionnaire about basic knowledge of mTBI	No effect of the intervention on symptom severity or attributions. Subgroup analyses suggested the benefit of web-based intervention in those receiving concurrent mental health treatment and in those participants with the greatest time since injury (>1 year after mTBI).	-	A
Boussi-Gross et al., 2013 [32]	Cross-over	44 y.o. 57% female	Vehicle accident = 38; falls = 7; object hit = 6; pedestrian accident = 3; assault = 2	1–5 years	To test the effectiveness of Hyperbaric Oxygen Therapy (HBOT) in improving brain Function and quality of life in mTBI patients suffering from chronic neurocognitive impairments.	56	-Cognition: Information Processing Speed index, attention-related index, memory-related index, executive functions index, Mindstreams battery -Quality of life: EQ-5D -Brain imaging: SPECT	Significant improvements were demonstrated in cognitive function and quality of life in both groups following HBOT.	+	A
Cifu et al., 2014 [34]	RCT	23.2 y.o. 100% male	Military	3 months–3 years	To evaluate the effects of hyperbaric oxygen therapy (HBOT) on persistent post-concussion symptoms.	60	-PCS: RPCSQ -PTSD: Post-traumatic Disorder Checklist-Military version	No effect on post-concussion symptoms after mTBI when compared with a control treatment.	-	A
Cooper et al., 2016 [24]	RCT	31 y.o. 93% male	Military	3–24 months	To compare different cognitive rehabilitation (CR) interventions (psycho-education, computer-based CR, therapist-directed manualized CR, and integrated therapist-directed CR combined with cognitive-behavioral psychotherapy) for mTBI.	126	-Cognition: PASAT -Mood: Symptom Checklist–90 Revised -Other: Key Behaviors Change Inventory	Equivalent improvement on the primary outcome (Paced Auditory Serial Addition Test). Improvements in primary outcome measures during treatment were maintained at follow-up with no differences among arms.	-	A
Harch et al., 2020 [31]	Single-blinded Cross-over	42 y.o. 56% female	21% Military	>6 months	To assess whether 40 150 kPa HBOT could improve cognitive symptoms of PCS after mTBI.	63	-Behavior: Neurobehavioral Symptom Inventory -Substance abuse: Michigan Alcohol Screening Test, Drug Abuse Screening Test -PTSD: Post-Traumatic Stress Disorder Check List-Military or Civilian, Post-Traumatic Stress Disorder Check List-Military or Civilian -Mood: Hamilton Anxiety Scale (HAM-A) -Cognition: Test of Memory Malingering, Green Word Memory Test, Wechsler Test of Adult Reading, Wechsler Adult Intelligence Scale or Wechsler Abbreviated Scale of Intelligence, Wechsler Memory Scale, Rey Auditory Verbal Learning Test Delayed Recall, Benton Visual Retention Test, Stroop Test, Controlled Oral Word Association Test, Category Fluency Test (Animals Test), and Automated Neuropsychological Assessment Metrics -Sleep: Pittsburgh Sleep Quality Index -Quality of life: QOLIBRI	HBOT participants improved significantly in the Neurobehavioral Symptom Inventory, Memory Index, Automated Neuropsychological Assessment Metrics, Hamilton Depression Scale, Hamilton Anxiety Scale, Post-Traumatic Stress Disorder Checklist, Pittsburgh Sleep Quality Index, and Quality of Life after Brain Injury compared to the control group, with the persistence of the improvement at 2 months.	+	A
Johansson et al., 2015 [25]	RCT	41 y.o. 59% female	Not described	>6 months	To assess the effect of Methylphenidate in two different dosages on mental fatigue, pain, and cognition.	51 (4 with moderate TBI)	-Cognition: Digit Symbol Coding from the WAIS-III; Digit Span; the Trail Making Test A and B	Significant effect on fatigue and processing speed (but not on pain) with a dose-dependent effect.	+	A
Johansson et al., 2017 [26]	Follow-up study of RCT	39.7 y.o. 60% female	Not described	>6 months	Thirty participants who had reported positive effects with methylphenidate during the initial phase of this follow-up study were treated with methylphenidate for a further six months.	30 (2 moderate TBI)	-Fatigue: Mental Fatigue Scale -Pain: VAS - Depression and anxiety: Comprehensive Psychopathological Rating Scale -Cognition: Digit Symbol Coding, Digit Span, TMT parts A and B	After a six-month follow-up, the effects of determined by the Mental Fatigue Scale, depression, anxiety, and cognitive function (processing speed, attention, working memory), were significantly improved compared to the baseline.	+	B
Johansson et al., 2020 [27]	5.5-year follow-up study of RCT	46 y.o. 59% female	Not described	>5 year	To assess the long-term effect of Methylphenidate. A comparison was made between those who had continued and those who had discontinued the treatment. The effect was also evaluated after a four-week treatment break.	17	-Fatigue: Mental Fatigue Scale -Pain: VAS - Depression and anxiety: Comprehensive Psychopathological Rating Scale -Cognition: Digit Symbol Coding, Digit Span, TMT parts A and B	Significant improvement in mental fatigue, depression, anxiety, and processing speed as compared to patients who discontinued treatment. Withdrawal produced significant deterioration in mental fatigue, depression, and anxiety, and a slower processing speed.	+	B
McAllister et al., 2011 [28]	Placebo-controlled double-blind RCT	27.2 y.o. 46% female	Falls or motor vehicle accidents	>1 month	To assess improvement of working memory by administration of an alpha-2 adrenergic agonist 1 month after injury.	13	-Cognition: N-back task in fMRI, WRAT-3, Reading subtest; WAIS-III, Block Design subtest; verbal memory (California Verbal Learning Test or California Verbal Learning Test-II; psychomotor speed (WAIS-III, Digit Symbol-Coding subtest; WM, executive, and attentional functioning (Trail Making Test, Parts A and B or Delis–Kaplan Executive Function System, Trail Making Test Conditions 2 and 4, D-KEFS Color-Word Interference Test, Controlled Oral Word Association Test, Paced Auditory Serial Addition Test, and Gordon Continuous Performance Test	Alterations in working memory after mTBI may be improved with the alpha-2 agonist.	+	B
Moussavi et al., 2019 [30]	Placebo-controlled double-blind RCT	49.5 y.o. 50% female	Falls, sports, and car accidents	>4.5 months	To assess if 4 sessions of 10 Hz rTMS applied on the dorso-lateral pre-frontal cortex could reduce post-concussion syndrome (assessed using the Rivermead Post Concussion Symptoms Questionnaire) after mTBI compared to sham stimulation.	22	-PCS: RPCSQ -Depression: Montgomery–Åsberg Depression Rating Scale -Electrovestibulography recording	Participants who had a more recent injury (<one year) and who received active rTMS showed significant improvements compared to those who received sham and to those with a longer duration of injury (>14 months) who received active rTMS.	+	A
Novakovic et al., 2020 [23]	Single-blinded controlled RCT	45.3 y.o. 12% female	military	>6 months	To assess the efficacy of cognitive rehabilitation of executive functions using Goaloriented attention self-regulation training (GOALS) in veterans with mTBI and PTSD.	40	-Cognition: Auditory Consonant Trigrams, the Letter Number Sequencing subtest from the Wechsler Adult Intelligence Scale, Third Edition, Color-Word Interference Test Inhibition task from the Delis-Kaplan Executive Function System, Trail Making Test-Part B, Design Fluency-Switching, Verbal Fluency Switching Total Correct (DKEFS), Color-Word Interference Test Inhibition- Switching (DKEFS), Digit Vigilance Test, Hopkins Verbal Learning Test—Revised, Brief Visual Memory Test—Revised, Goal Processing Scale -Daily and emotional functioning: Mayo-Portland Adaptability Inventory, Goal Processing Questionnaire -Psychology: Profile of Mood States, Beck Depression Inventory-II, PTSD Checklist, Military Version	Participants of the group benefiting from the GOALS rehabilitation improved a composite score of neuropsychological tests assessing executive and attentional functions.	+	A
Rytter et al., 2018 [19]	Controlled RCT	18–29 y.o. = 26.7% 30–43 y.o. = 46.7% >44 y.o. = 26.7% 64% female	Fall = 47%; sports accidents = 33%	>6 months	RCT to compare the effectiveness of a specialized, interdisciplinary rehabilitation (S-REHAB) with standard care (STAND) for people with persistent post-concussive symptoms	89	-PCS: RPCSQ -Headache: Headache Impact Test -Depression: Major Depression Inventory -Fatigue: Multidimensional Fatigue Inventory-20 -Quality of life: SF-36	Significant reduction in symptoms measured by the Rivermead Post-Concussion Symptoms Questionnaire compared to the STAND immediately post-treatment and at 6 months of follow-up.	+	A
Storzbach et al., 2016 [20]	RCT	35 y.o. 6% female	Military	Not indicated	To evaluate the efficacy of group-based Compensatory Cognitive Training (120 min group sessions per week, 10 weeks) in veterans with mTBI compared to usual care.	119	-Questionnaires: Prospective-Retrospective Memory Questionnaire, Multiple Sclerosis Neuropsychological Screening Questionnaire—Patient Version, Memory Compensation Questionnaire, Portland Cognitive Strategies Scale 2.0, Neurobehavioral Symptom Inventory, the Wide Range Achievement Test- Reading subtest –Cognition: Hopkins Verbal Memory Test—Revised, Wechsler Adult Intelligence Scale—4th Edition, Digit Span Subtest and Digit Symbol Subtest (WAIS-IV), Delis-Kaplan Executive Function System, Trails Sub-test and Verbal Fluency Subtest -PTSD: PTSD Checklist—Military Version -Depression: Beck Depression Inventory, Second Edition -Quality of Life: Satisfaction with Life Scale -Adaptive Functioning: University of California San Diego (UCSD) Performance-Based Skills Assessment, Brief Version	Training in compensatory cognitive strategies facilitates behavioral change (use of cognitive strategies) as well as both subjective and objective improvements in targeted cognitive domains.	+	A
Thastum et al., 2019 [22]	Unmasked RCT	22.9 y.o. 79% female	Traffic accidents = 32%; falls = 26%; sport = 21%	2–6 months	To test an interdisciplinary, individually tailored intervention of 8 weeks duration based on a gradual return to activities and principles from cognitive behavioral therapy (GAIN).	112	-PCS: RPCSQ -2 subscales from The Behavioral Responses to Illness Questionnaire -Quality of life: The Quality of life after Brain Injury–Overall scale, SF-36 -Psychology: Symptom Checklist-90-Revised, 10-item Perceived Stress Scale -Executive functions: Behavior Rating Inventory of Executive Function–Adult version -Patient Global Impression of Change	Patients treated with GAIN plus enhanced usual care reported a significantly larger reduction in PCS than patients receiving enhanced usual care alone.at 3-month follow-up.	+	A
Theadom et al., 2013 [29]	Double-blind placebo-controlled Phase II RCT	44 y.o. 57% female	Motor vehicle accidents = 33.3%; falls = 33.3%; sports-related injury = 10.0%	3–12 months	To investigate the safety, compliance, and efficacy of Enzogenol (pine extract) for improving cognitive functioning in people 3–12 months following mild TBI.	62	-safety and tolerability -Cognition: CFQ, digit span subtest of the Wechsler Adult Intelligence Scale Version 3, Arithmetic WAIS Version 4, Letter Number Sequencing sub-tests of the WAIS Version 4, California Verbal Learning Test -PCS: RPCSQ -Anxiety and mood: HADS	Administration of Enzogenol during 6 weeks is well-tolerated and may reduce self-perceived cognitive failures in patients 3–12 months post-mild TBI.	+	B
Tiersky et al., 2005 [21]	Single-blind randomized, wait-listed controlled trial, with repeated measures and multiple baselines.	47 y.o. 55% female	Motor-vehicle accidents = 65%; falling objects = 15%; falls = 10%, sports-related accidents = 5%	1–20 years (mean 6.25 y)	To test the effect of a neuropsychologic rehabilitation program, including psychotherapy and cognitive remediation. Treated patients received both individual cognitive-behavioral psychotherapy and cognitive remediation 3 times a week for 11 weeks.	20 (including 2 with moderate TBI)	-Cognition: Paced Auditory Serial Addition Task, Rey Auditory Verbal Learning Test, ACFI, the Attention Questionnaire. -Psychosocial and affective functioning: Coping Response Inventory and SCL- 90R. -Community participation: Community Integration Questionnaire.	Significant reduction in anxiety and depression and improvement in divided attention at 1 and 3 months follow-up. However, no significant improvement in community integration scores.	+	A
Walker et al., 2014 [33]	Sham-controlled double-blind RCT	23.2 y.o. 0% female	Military	3–36 months	To assess the effect of HBOT on persistent post-concussion symptoms.	60	-Cognition: D-KEFS letter fluency, Trail Making Test part B, Stroop Color-Word, CPT-II detectability, PASAT, WAIS-III Working Memory Index, CVLT, Benton Visual Memory Test	No immediate post-intervention beneficial effect of exposure to hyperbaric oxygen.	-	B
Wolf et al., 2012 [80]	Sham-controlled RCT	28.3 y.o. 4% female	Military	3–71 months	To evaluate the effect of HBOT on post-concussive symptoms after mild TBI.	50	-Cognition: ImPACT Immediate Post-Concussion Assessment and Cognitive Testing -PTSD: PCL-M	No significant differences in cognitive disabilities (on PCL-M composite score nor on the ImPACT total score).	-	A

**Table A2:** Sleep (four articles).

First Author, Year of Publication	Study Design	Age (Mean ± Standard Deviation) and Gender	Etiology of mTBI	Delay between mTBI and Inclusion	Main Aim	Number of Participants	Outcomes and Tools	Results	+ If Main Result Is Significant, − If Not	Incog Recommendation Level
Killgore et al., 2020 [39]	Double-blind RCT	23 y.o. 53% female	Not described	1–18 months (mean 6 months)	To evaluate the effect of blue-wavelength light on cognitive functions at 1–18 months from mTBI.	32	-Sleep: duration, circadian phase shift, subjective and objective sleepiness -Cognition: psychomotor vigilance test, RBANS (battery) -Functional Connectivity -White matter axonal integrity. Each of these was assessed at the baseline week or visit and again during the final week or follow-up visit.	Participants of the experimental group significantly improved phase-advanced sleep timing, reduced daytime sleepiness, and improved their processing speed during the Tower of London task compared to the control group (Amber light) but not their results at a brief neurocognitive performance battery (RBANS).	+	A
Raikes et al., 2020 [40]	Placebo-and double-blind controlled RCT	26 y.o. 63%	Motor vehicle accident = 50%	<18 months	To assess the effect of 30 min morning blue light therapy during 6 weeks on sleep in mTBI participants at 1–18 months from TBI.	35	-Sleep: Epworth Sleepiness Scale, Pittsburgh Sleep Quality Index, Functional Outcome of Sleep Questionnaire, daily sleep onsets and offsets, total time in bed, total nighttime sleep time, sleep efficiency and normalized wake after sleep onset -Depression: Beck Depression Inventory-II -PCS: RPCSQ	The authors found lower Epworth Sleepiness Scale, Beck Depression Inventory-II, Rivermead Post-Concussion Symptom Questionnaire chronic, and somatic symptoms in the experimental group. These participants had greater total sleep time and better scores on sleep questionnaires.	+	A
Theadom et al., 2017 [38]	RCT (pilot study)	35.9 y.o. 63% female	Motor vehicle accident = 50%; hit by object = 21%; other = 29%	3–36 months	To explore the feasibility and potential efficacy of online interventions for sleep quality following mTBI.	24 (1 moderate TBI in each group)	-Sleep: Pittsburgh Sleep Quality Index, record of time taken to fall asleep, sleep efficiency, time spent awake after sleep onset, and number of awakenings -PCS: RPCSQ -Cognition: CNS Vital Signs online neuropsychological assessment -Quality of life: Quality of Life after Brain Injury questionnaire	Significant reductions in sleep disturbance in comparison to controls post-intervention. There were no significant group differences in objective sleep quality, cognitive functioning, post-concussion symptoms, or quality of life.	-	B
Vuletic et al., 2016 [37]	Single-blind RCT	29.3 y.o. 6% female	Military	<2 years	Randomized trial to evaluate the effect of telephone-based problem-solving treatment (PST) on sleep quality in active duty post-deployment service members with mild traumatic brain injury.	356	-Sleep: Pittsburgh Sleep Quality Index -PCS: RPCSQ -Psychological symptoms: Behavioral Symptoms Inventory-18, PHQ-9 -PTSD: PTSD Checklist-Military Version -Pain: EQOL4, EQOL4 -Symptoms’ impact: SF-12, Sheehan Disability Scale	PST significantly improved sleep quality at 6 months but not at 12 months.	-	A

**Table A3:** Treatment of somatic complaints and fatigue (15 articles).

First Author, Year of Publication	Study Design	Age (Mean ± Standard Deviation) and Gender	Etiology of mTBI	Delay between mTBI and Inclusion	Main Aim	Number of Participants	Outcomes and Tools	Main Result	+ If Main Result Is Significant, − If Not	Incog Recommendation Level
Choi et al., 2018 [48]	Randomized controlled feasibility study	42.6 y.o. 50% female	Not described	Chronic (mean 15.7 months)	To assess if 10 sessions of 10 Hz rTMS applied on the primary motor cortex of the affected hemisphere could reduce central pain after mTBI (assessed using a numerical rating scale NRS) compared to sham stimulation.	12	-Pain: NRS -Quality of life: SF-36 (Physical and Mental Component Scores)	The NRS score was significantly lower in the experimental group with rTMS than in the control group immediately after treatment, at one, two, and four weeks.	+	B
Cifu et al., 2014 [35]	Sham-controlled blinded RCT	23.3 y.o. 0% female	Military	3 months–3 years	To evaluate the effects of hyperbaric oxygen on eye-tracking abnormalities in males after mTBI.	60	Eye-tracking analyses	No effect on post-concussive eye movement abnormalities after mTBI when compared with a sham-control.	-	A
Esterov et al., 2021 [51]	RCT	44 y.o. 65% female	Motor vehicle accidents = 70%; falls = 20%; sports = 10%	3–88 months	To assess the effect of osteopathic manipulative treatment on headaches after mTBI.	26	-Pain: headache scores on a Visual Analog Scale (VAS) and scores on the six-item Headache Impact Test (HIT-6)	Significant improvement of the VAS in the intervention group but not in the HIT-6.	+	B
Jensen et al., 1990 [50]	RCT	31 y.o. 63% female	Not described	9–12 months post-injury	To test if manual therapy (two sessions) on the neck could reduce post-traumatic headaches. The control group was treated with cold packs on the neck.	23	-Pain: VAS, use of analgesics -Frequency of associated symptoms -Neck motion	Significant reduction of the pain index in the treatment group two weeks after the end of treatment, but this effect was no more statistically significant five weeks later.	-	B
Kjeldgaard et al., 2014 [49]	RCT	34 y.o. gender not described	Traffic accident = 45%	Mean time since injury: 27 months	To evaluate the effect of a group-based Cognitive Behavioral Therapy (CBT) intervention in relation to headache, pain perception, psychological symptoms, and quality of life in patients with CPTH.	90	-Headache: diary -PCS: Rivermead Post Concussion Symptoms Questionnaire -Quality of life: SF-36 -Psychology: SCL-90-R	A nine-week CBT had no effect on headache and pressure pain thresholds in comparison to a waiting list group.	-	A
Kleffelgaard et al., 2019 [41]	Blinded RCT	39 y.o. 70% female	Traffic accidents = 23%; falls = 9%; violence = 64%; sport = 9%	Mean 3.5 months	To investigate the effects of 8 weeks of group-based vestibular rehabilitation in patients with dizziness after mild traumatic brain injury.	65	-Dizziness-related disability: Dizziness Handicap Inventory, Vertigo Symptom Scale-Short Form -Mobility: High-Level Mobility Assessment Tool for TBI -PCS: RPCSQ -Psychology: HADS -Balance: Balance Error Scoring System	Statistically significant mean differences in Dizziness Handicap Inventory at 2.2 ± 0.8 months compared to control. No significant difference 2 months after the end of rehabilitation.	+	A
Kolakowsky-Hayner et al., 2017 [55]	Single-blinded crossover	42.7 y.o. 61% female	Vehicular trauma = 53.7%; violence = 6.5%; sports = 8.1%; fall/hit = 12.2%; pedestrian = 8.9%	At least 6 months after TBI	To evaluate the impact of a graduated physical activity program on fatigue after TBI.	123	-Fatigue: Global Fatigue Index, Barrow Neurological Institute Fatigue Scale Overall Severity Index Score, Multidimensional Fatigue Inventory	Less reported fatigue at the end of the intervention (24 weeks) and after a washout period (36 weeks).	+	B
Leung et al., 2016 [45]	RCT	41 y.o. 12.5% female	Military	Chronic (33–580 months)	A sham-controlled randomized study to assess the effect of repetitive transcranial magnetic stimulation (rTMS, 10 Hz, 2000 pulses, 20 trains, one-sec inter-train interval) at the left motor cortex) in reducing mTBI-related headaches.	24	-Headache: diary -Cognition: Conner’s Continuous Performance Test -Mood: Hamilton Rating Scale for Depression -PTSD: Mississippi scale for PTSD -Global Pain: Brief Pain Inventory	There was a significant reduction in persistent headache intensity and debilitating headache exacerbation in the real treatment group at 1 and 4 weeks post-treatment.	+	B
Leung et al., 2018 [46]	RCT	34.1 y.o. 21% female	Military	21–367 months	To assess if 4 sessions of 10 Hz rTMS applied on the left prefrontal cortex could reduce headaches after mTBI (assessed using a diary) compared to sham stimulation.	44	-Headache: diary (sum of headache NRS score or sum of the duration of the HA/number of days) -Cognition: Conner’s Continuous Performance Test, the Wechsler Adult Intelligence Scale (WAIS-IV), Hopkins Verbal Learning Test, Stroop Test, -Mood: Hamilton Rating Scale for Depression -Global pain: Brief Pain Inventory –PTSD: Clinician-Administered PTSD Scale (CAPS)	The group benefiting from rTMS showed a significant decrease in the average NRS at 1 and 4 weeks and a significant decrease in the prevalence of persistent headaches at 1 and 4 weeks. The experimental group showed a significant improvement in the Hamilton Rating Scale for Depression score at 1-week.	+	A
Meehan et al., 2019 [44]	Sham-controlled double-blind RCT	32.8 y.o. 1% female	Military	3 months–5 years	To describe the effect of HBO2 sessions during 3 months on balance function in military service members.	71	-Balance: NeuroCom Computerized Dynamic Posturography, Cervical Vestibular Evoked Myogenic Potential, Ocular Vestibular Evoked Myogenic Potential, tandem gait, Romberg, Sharpened Romberg, Berg Balance Scale -Anxiety and Mood: Beck Anxiety Inventory, Center for Epidemiologic Study–Depression Scale -PTSD: PTSD Checklist-Civilian Version, Structured Clinical Interview for the DSM-IV, PTSD Module	Minimal significant trend on balance after HBO2. Those with affective symptoms had the most improvement in postural control and otolith function.	-	A
Schneider et al., 2014 [43]	Single-blinded RCT	15 y.o. 42% female	Sport	8–276 days (average: 53 and 47 days in the treatment and control group, respectively)	To determine if a combination of vestibular rehabilitation and cervical spine physiotherapy decreased the time until medical clearance in individuals with prolonged PCS (weekly sessions for 8 weeks)	31	-Number of days from treatment initiation until medical clearance to return to sport -Pain: 11-point Numeric Pain Rating Scale score -Balance: Activities-specific Balance Confidence Scale, Functional Gait Assessment -Dizziness; Dizziness Handicap Index, Other: SCAT2 Dynamic Visual Acuity, Head Thrust Test, Modified Motion Sensitivity Test, Cervical Flexor Endurance and Joint Position Error test	Treated patients showed a decreased time to medical clearance to return to sport within 8 weeks compared to the control group receiving conventional physiotherapy	+	A
Stilling et al., 2019 [47]	RCT	36.0 y.o. 90% female	Vehicle accidents = 47%; sports = 32%; falls = 11%; other = 11%	3 months–5 years	To assess the effect of 10 sessions of rTMS (10 Hz, 600 pulses, 70% resting motor threshold amplitude, on the left dorsolateral prefrontal cortex) on headaches at 1 month in participants with mTBI and persistent post-traumatic headaches.	20	-Headache: diary, headache impact test–6 -PCS: RPCSQ, British Columbia post-concussion symptom inventory (BC-PSI) -Cognition: Montreal cognitive assessment -Quality of life: quality of life after brain injury questionnaire (QOLIBRI) -Health: participant health questionnaire-9 –Anxiety: generalized anxiety disorder scale-7 –PTSD: post-traumatic stress disorder checklist for DSM-V	The authors found a significant overall time effect for average headache severity and frequency (based on a diary) 1 month after treatment.	+	B
Straudi et al., 2017 [42]	RCT	36 y.o. 19%	Not described	>12 months	A randomized study to test the effects of video game therapy compared with a balance platform therapy on balance, mobility, and selective attention in chronic traumatic brain injury patients.	21	-Balance: CB&M, Unified Balance Scale, Timed Up and Go test -Attention: Go/No Go task -Static balance: force plate	Both groups improved in Community Balance and Mobility Scale scores, but only the video game therapy group increased on the Unified Balance Scale and Timed Up and Go test.	+	B
Thiagarajan and Ciuffreda, 2013 [52]	Cross-over design	29 y.o. % of females not described	Not described	1–10 years	To evaluate the vergence before and after vergence-based OMT in individuals who reported near work-related symptoms of an oculomotor nature after mTBI.	12	-Binocular central fixation, saccadic gain, saccadic latency, saccade ratio	Vergence-based oculomotor rehabilitation was effective in individuals with mTBI who reported near work-related symptoms: reduction in the horizontal fixational error, increase in horizontally and vertically saccadic gain, and decrease in the saccade ratio for reading.	+	B
Thiagarajan et al., 2014 [53]	Cross-over design	29 y.o. % of females not described	Not described	1–10 years	To evaluate the effect of oculomotor-based vision rehabilitation (OBVR) during 6 weeks (2 sessions per week) in mTBI.	12	-Near point of convergence, near point of accommodation, reading eye movements, saccade ratio, reading rate, Convergence Insufficiency Symptom Survey -Visual attention: Visual search and Attention test	OBVR had a strong positive effect on oculomotor control, reading rate, and overall reading ability, with an improvement of over 80% of the abnormal parameters.	+	B

**Table A4:** Early interventions (18 articles).

First Author, Year of Publication	Study Design	Age (Mean ± Standard Deviation) and Gender	Etiology of mTBI	Delay between mTBI and Inclusion	Main Aim	Number of Participants	Outcomes and Tools	Results	+ If Main Result Is Significant, − If Not	Incog Recommendation Level
Andersson et al., 2007 [65]	RCT	32 y.o. 41% of females in the intervention group, 33% in the control group	Traffic accidents = 24%; falls = 45%; blow = 25%	2–8 weeks	To assess if an early individualized, tailored, multidisciplinary outpatient rehabilitation program of selected patients with mTBI may reduce long-term (1 year) outcomes.	395	-PCS: Post-Concussion Symptoms Questionnaire -Life satisfaction: Life Satisfaction Questionnaire -Activity, participation and quality of life: Community Integration Questionnaire, Short-Form Health Survey 36 items, Interest Checklist, Role Checklist, Job Satisfaction Checklist	No statistically significant difference between the groups in terms of PCS and quality of life.	-	A
Audrit et al., 2021 [71]	Parallel groups (experimental and wait-list control)	39 y.o. 60% female	Motor vehicle accidents = 16%; sports = 16%; other = 68%	1–3 months	To assess the effect of a psycho-educative and counseling intervention named SAAM (Sleep/fatigue, Attention, Anxiety/mood, Memory) on PCS assessed using the Rivermead Post-Concussion Symptoms Questionnaire and on anxiety, depression, sleep, fatigue, memory, attention, and participation.	25	-PCS: RPCSQ -Sleep: Pittsburgh Sleep Quality Index -Anxiety and mood: Hospital Anxiety and Depression Scale -Fatigue: Multidimensional Fatigue Inventory -Cognition: attention and memory neuropsychological battery -Participation: Community Integration Questionnaire	No significant group x time interaction for the Rivermead Post-Concussion Questionnaire.	-	A
Bell et al., 2008 [58]	RCT with blinded assessment	33 y.o. 35% female	Vehicle accidents = 55%; assault = 14%; sports = 5%; falls = 15%; other = 11%	<3 months	To determine whether focused, scheduled telephone counseling during the first 3 months after MTBI decreases symptoms and improves functioning at 6 months.	366	-PCS: Head Injury Symptom Checklist -Quality of life: Short Form Health Survey-12, Modified Perceived Quality of Life -Health, anxiety, mood: Patient Health Questionnaire (PHQ)-Depression and Panic/Anxiety	Significantly better 6-month outcome for symptoms and daily functioning but no difference in general health outcomes.	+	A
Caplain et al., 2019 [59]	RCT	37 y.o. 63% female	Attacks = 33%; falls = 39%; sports = 0%; vehicle accidents = 19%	<1 month	To assess an early multidimensional intervention associating psycho-education and cognitive rehabilitation in patients presenting high-risk factors for persistent PCS. The control group received psycho-education alone.	80	-PCS: DSM-IV criteria -Work: cessation, resumption -Cognition: Digit span forward and backward (Wechsler Memory Scale), Mental control (MEM III, Wechsler Memory Scale), Letter-number sequences (MEM III, Wechsler Memory Scale), Trail Making Test, Parts A and B, Stroop test, Paced Auditory Serial Addition Test (PASAT), D2 Test of Attention, categorical and phonemic verbal fluency, Rey 15-item memory test -Quality of life: Quality of Life after Brain Injury (QOLIBRI) questionnaire -Anxiety and mood	At the 6-month follow-up, the proportion of patients with PCS was significantly lower in the treated group compared to the controls.	+	A
Chen et al., 2013 [72]	Double-blind placebo-controlled RCT	45 y.o. 34% female	Not described	<1 day	To investigate how cerebrolysin therapy enhances cognitive recovery for mTBI patients.	32	-Cognition: MiniMental State Examination (MMSE), Cognitive Abilities Screening Instrument (CASI)	Improvement of CASI score at 12 weeks, especially concerning long-term memory and drawing function, but not the MMSE.	+	B
De Kruijk et al., 2005 [63]	RCT	37 y.o. 44% female	Traffic accidents = 48%; work = 18%; violence = 8%; sports = 10%	<10 days	To evaluate the effect of bed rest on the severity of post-traumatic complaints after mTBI.	107	-PCS: 16 post-traumatic complaints assessed on a visual analogic scale -Quality of life: SF-36	No significant differences between bed rest and no bed rest conditions on recovery.	-	A
Ghaffar et al., 2004 [64]	RCT	32 y.o. 33% female	Motor-vehicle accidents = 48%	<one week	To determine whether multidisciplinary treatment of mTBI improves neurobehavioral outcomes at 6 months after injury.	191	-PCS: Rivermead Post-Concussion Disorder Questionnaire -Activity: Rivermead Follow-up Questionnaire -Anxiety and mood: 28-item General Health Questionnaire -Cognition: Stroop Color-Word Test, Symbol-Digit Modalities Test, Paced Visual Serial Addition Task, Simple Reaction Time, Choice Reaction Time, Hopkins Verbal Learning Test-Revised, the Vocabulary subtest of the Wechsler Adult Intelligence Scale—Third Edition (WAIS-III), the Letter–Number Sequencing subtest of the WAIS-III, the Matrix-Reasoning subtest of the WAIS-III	At 6 months after injury, there was no difference between the treatment group and control on any of the tests administered. However, in subjects with a psychiatric history, the provision of treatment was associated with significantly less depressive symptoms (GHQ subscale) 6 months after injury compared with untreated individuals.	-	A
Heskestad et al., 2010 [66]	RCT			2 weeks after the injury	To evaluate the effect of an educational intervention on outcome after minimal, mild and moderate head injury (cognitive-oriented counseling, advice, the proposition of coping strategies, information, and reassuring) through one single consultation. Follow-up at 3 and 12 months. Outcome measures: interviews/symptoms, Beck Depression Inventory, Epworth Sleepiness Scale and the Fatigue Severity Scale.	326	-Mood: Beck Depression Inventory -Vigilance: Epworth Sleepiness Scale -Fatigue: Fatigue Severity Scale -Quality of life: SF-36	No statistically significant differences between the intervention group and the control group. However, only 15% of patients completed the study.	-	B (high number of drop-out)
Matuseviciene et al., 2013 [67]	RCT	39 y.o. 50% female	Falls = 42%; car or bicycle accidents = 17%; sports = 16%; assault = 5%	Early: 14–21 days	To investigate the effect of an early intervention visit in addition to written information and treatment as usual for patients with an estimated high risk for persisting disability after mTBI.	97	-PCS: RPCSQ -Anxiety and mood: HADS	No additional effect on symptom level at 3 months after mTBI compared to the usual treatment group.	-	A
Mittenberg et al., 1996 [56]	RCT with blinded 6-month assessment	46 y.o. 31% female	Motor vehicle accidents = 59%; falls = 21%	Very early intervention (prior to discharge from the hospital)	To test the effectiveness of a structured cognitive-behavioral procedure for the prevention of PCS. Treatment based on a printed manual and consultation prior to discharge (psycho-education, information, techniques for reducing symptoms, and instructions for gradual resumption of premorbid activities).	58	-PCS: duration, number of symptoms, frequency of initial symptoms, days per week symptomatic at 6 months, and the severity of average symptoms at 6 months	6-month outcome: treated patients reported significantly shorter symptom duration, fewer symptoms, fewer symptomatic days, and lower severity levels.	+	A
Moore et al., 2014 [62]	Non-randomized quasi-experimental study	39 y.o. 27% female	Assault = 25%; bicycle or motor vehicle accidents = 38%; falls = 20%	<24 h	To determine the acceptability and effectiveness of Emergency Department Social Work Intervention for mTBI (SWIFT-Acute) on alcohol use, community functioning, depression, anxiety, post-concussive symptoms, post-traumatic stress disorder, and service use.	64	-Alcohol Use Disorders Identification Test -Participation: Community Integration Questionnaire -Qualitative acceptability survey -Health: patient Health Questionnaire-4 -PCS: RPCSQ -PTSD: Post-traumatic Stress Disorder Checklist-Civilian	3-month outcome: Preliminary evidence of effectiveness in reducing alcohol use and preventing functional decline. No statistically significant differences were found in other measures.	+	B
Ponsford et al., 2002 [57]	Pseudo-RCT (participants were alternately assigned to one of two groups)	26 y.o. gender not described	Motor vehicle accidents = 27%; falls = 18%; cycling accidents = 7%; assaults = 18%; sport = 23%	One week	To evaluate the impact of an information booklet outlining the symptoms associated with mTBI and suggestions for coping strategies.	202	-PCS: post-concussion syndrome checklist -Psychological adjustment: symptom checklist-90-revised -Stress: Holmes–Rahe survey of recent experiences -Cognition: national adult reading test (NART),13 four-choice reaction times (decision time), the Wechsler adult intelligence scale (reading) (WAIS-R) digit span and digit symbol subtests, the 2.4 and 2.0 s pacings of the paced auditory serial addition task (PASAT), the speed of comprehension subtest from the speed and capacity of language processing test, and the Rey auditory-verbal learning test	Patients in the intervention group reported fewer symptoms and were less stressed three months after the injury.	+	A
Robertson et al., 2017 [73]	Placebo-controlled RCT	29 y.o. 33% female	Not described	<one week	To determine if the administration of atorvastatin 7 days after mTBI would improve neurological recovery.	52	-PCS: RPCSQ -adverse effects	No significant differences in neurological recovery after mTBI using atorvastatin versus a placebo at 3 months.	-	B
Scheenen et al., 2017 [70]	RCT	41 y.o. 55% female	Not described	4–6 weeks	Effectiveness of early cognitive behavioral intervention (CBTi) compared to telephonic counseling (TC) in at-risk mTBI patients.	91	-Return-to-work -Functional Outcome: Glasgow Outcome Scale-Extended -PCS: Head Injury Symptom Checklist -Coping: Utrechtse Coping List -Anxiety and mood: Hospital Anxiety and Depression Scale	No difference in return-to-work rate, coping styles and levels of anxiety, and depression at 3, 6, and 12 months. Patients in the TC group presented a more favorable outcome at 12 months and fewer complaints at 3 and 12 months.	+	A
Silverberg et al., 2013 [60]	Masked RCT	39 y.o. 61% female	Motor vehicle accidents = 43%; falls = 32%; bicycle accidents = 21%; sport = 4%	<6 weeks	To assess the effect of early CBT, in addition to treatment as usual, on patients at risk for chronic PCS.	28	-PCS: RPCSQ -Daily functioning: Mayo-Portland Participation Index -Illness Perception Questionnaire—Revised -Anxiety and mood: Hospital Anxiety and Depression Scale	Masked outcome assessment was conducted at a 3-month follow-up. Significantly fewer treated participants had a diagnosis of PCS at follow-up compared to the controls. Treatment effect size was moderate for post-concussion symptoms.	+	A
Suffoletto et al., 2013 [61]	RCT	30 y.o. 56% female	Not described	Early < 14 days	Randomized controlled study with 14-day follow-up to assess the efficacy of text messaging-based education and behavioral support on severe post-concussive symptoms.	43	-PCS: RPCSQ, DSM-IV criteria -PTSD: PTSD screen -Anxiety and mood: PHQ-4 -self-medication for pain -perception of the text messaging program	A trend toward lower reports of headaches, concentration difficulty, irritability, and anxiety in the group receiving text messages.	+	B
Varner et al. (2017) [69]	RCT	35.2 y.o. 64% female	Assault = 7%; sport = 9%; bicyle or motor vehicle accidents = 26%; falls = 41%	<24 h	To determine if patients receiving cognitive rest and graduated return to usual activity discharge instructions had a decrease in PCS 2 weeks after injury.	118	-PCS: Post-Concussion Symptom Score -Number of missed days of school or work -Repeat visits to a healthcare provider	No significant group difference at 2- and 4-week assessment regarding post-commotional symptoms.	-	A
Vikane et al., 2017 [68]	RCT	32 y.o. 39% female	Traffic accidents = 29%; falls = 37%; assault = 18%; sport = 16%	6–8 weeks after mTBI	To evaluate the efficacy of a multidisciplinary outpatient follow-up program compared to follow-up by a general practitioner for patients being at-risk or sick-listed with persistent post-concussion symptoms two months after a mild traumatic brain injury.	151	-Return-to-work: delay -PCS: RPCSQ -Disability: Glasgow Outcome Scale Extended -Patient’s Global Impression of Change -Anxiety and mood: Hospital Anxiety and Depression Scale	No improvement in return-to-work and fewer post-concussion symptoms in the intervention compared to the control Group at 12 months.	-	A
